# Does addition of craving management tools in a stop smoking app improve quit rates among adult smokers? Results from BupaQuit pragmatic pilot randomised controlled trial

**DOI:** 10.1177/20552076211058935

**Published:** 2021-11-23

**Authors:** Aleksandra Herbec, Lion Shahab, Jamie Brown, Harveen Kaur Ubhi, Emma Beard, Alexandru Matei, Robert West

**Affiliations:** 1 Department of Behavioural Science and Health, 4919 University College London, UK; 2 Department of Clinical, Educational and Health Psychology, UCL Centre for Behaviour Change, 4919 University College London, UK; 3 UCL Tobacco and Alcohol Research Group (UTARG), 4919 University College London, UK; 4 Department of Clinical, Educational and Health Psychology, 4919 University College London, UK; 5 Bupa Centre Medical, UK; 6 Department of Computer Science, 4919 University College London, UK

**Keywords:** smoking, cessation, smartphone, cravings, randomised controlled trial

## Abstract

**Objectives:**

Delivery of craving management tools via smartphone applications (apps) may improve smoking cessation rates, but research on such programmes remains limited, especially in real-world settings. This study evaluated the effectiveness of adding craving management tools in a cessation app (BupaQuit).

**Methods:**

The study was a two-arm pragmatic pilot parallel randomised controlled trial, comparing a fully-automated BupaQuit app with craving management tool with a control app version without craving management tool. A total of 425 adult UK-based daily smokers were enrolled through open online recruitment (February 2015–March 2016), with no researcher involvement, and individually randomised within the app to the intervention (*n* = 208) or control (*n* = 217). The primary outcome was self-reported 14-day continuous abstinence assessed at 4-week follow-up. Secondary outcomes included 6-month point-prevalence and sustained abstinence, and app usage. The primary outcome was assessed with Fisher's exact test using intent to treat with those lost to follow-up counted as smoking. Participants were not reimbursed.

**Results:**

Re-contact rates were 50.4% at 4 weeks and 40.2% at 6 months. There was no significant difference between intervention and control arms on the primary outcome (13.5% vs 15.7%; *p* = 0.58; relative risk = 0.86, 95% confidence interval = 0.54–1.36) or secondary cessation outcomes (6-month point prevalence: 14.4% vs 17.1%, *p* = 0.51; relative risk = 0.85, 95% confidence interval = 0.54–1.32; 6-month sustained: 11.1% vs 13.4%, *p* = 0.55; relative risk = 0.83, 95% confidence interval = 0.50–1.38). Bayes factors supported the null hypothesis (*B*[0, 0, 1.0986] = 0.20). Usage was similar across the conditions (mean/median logins: 9.6/4 vs 10.5/5; time spent: 401.8/202 s vs 325.8/209 s).

**Conclusions:**

The addition of craving management tools did not affect cessation, and the limited engagement with the app may have contributed to this.

## Introduction

Use of face-to-face and telephone-based smoking cessation support is low even when it is free at the point of access.^1^ Smartphone apps may appeal to smokers not willing to use these forms of support.^2^ In 2016, over 85% of the UK population had access to a smartphone.^3^ However, most cessation apps do not offer evidence-based support.^4–7^ Results of randomised controlled trials (RCTs)^8–10^ and observational studies^11–13^ have not yet provided clear evidence that such apps can aid cessation. Recently a decision-aid app improved self-reported quit rates over a control app version.^14^ The present pilot RCT assessed how far the inclusion of craving management tools (CMTs) in an app could improve cessation. Furthermore, in order to recreate for the participants a more authentic experience of app download, and to increase the generalisability of findings, the study involved limited contact with the researchers and low participant burden at enrolment, in contrast to earlier research.^8,9,12^

Cigarette craving can be defined as the experience of strong motivation (desire, need or urge) to smoke and is predictive of relapse.^15^ Several techniques reduce momentary cravings, including distraction, imagining pleasant experiences,^16^ relaxation,^17^ physical exercise^18^ and yogic breathing.^19^ Cessation interventions that include behaviour change techniques (BCTs)^20^ that reduce, or improve coping, with cravings appear to improve success rates.^20^ An advantage of apps, also over SMS texting, is that they could be used online and offline, present multimedia content, and thus offer an opportunity to deliver or prompt use of different CMTs when appropriate. In principle, such apps could improve quit rates.

The SF28 (‘SmokeFree28’) app is an existing app that supports smokers to be smoke-free for 28 days as the first step to long-term abstinence, through offering features to set up a quit date and monitor progress, as well as advice on cessation and medication use, and motivational content (http://www.sf28.co.uk/).^11,21^ SF28 is informed by PRIME theory of motivation, which postulates that quitting requires maintaining sufficiently high desire and capacity to override emerging impulses to smoke.^22^ In an observational study, SF28 produced self-reported short-term abstinence that was higher than would have been expected with unaided cessation.^11^ This app was also judged to contain all the evidence-based features that might be expected to improve cessation^23^ and achieved relatively high engagement rates (8.5 (SD = 9.0) mean logins).^11^ SF28 was therefore chosen as the basis for the development of a new app to evaluated specific CMTs. The new app was sponsored by the healthcare company Bupa and was called ‘BupaQuit’. The appearance of SF28 was redesigned while keeping the key logic, content, and the user flow was similar.

Two versions of BupaQuit were created: one with a set of CMTs, and the other without them. The control app was designed to be a minimal credible intervention (MCI),^24^ and to be similar to the intervention app in many respects (e.g. user journey, layout, core advice), but not to offer key intervention components. Provision of a true inactive control condition or a waitlist in smartphone research may be impossible given the availability of other free quit smoking apps, and so the control version of BupaQuit was judged to be fairer and a more realistic comparison for the trial. Using an MCI would also support enrolment and follow-up, and ensure comparable user experience and data collection across study arms. Furthermore, a poorly performing control app could lack credibility, damage the long-term reputation of BupaQuit program, undermine cessation rather than simply being ‘neutral’ and encourage users to seek alternative apps.^24^

### Aims

This study aimed to estimate the impact of the inclusion of CMTs within an app that was live on app stores on cessation and engagement levels, compared with an app version without those features, and to assess the feasibility of remote enrolment and follow-up procedures.

## Materials and methods

### Design

This study was a two-arm parallel double-blind pragmatic pilot RCT conducted remotely in the UK, with participants randomised automatically within the app (after registration) in 1:1 ratio to either the intervention or control app (random numbers generated using a standard JavaScript library). The study was prospectively registered on an international trial registry (ISRCTN10548241). Additional documentation is available on the Open Science Framework (OSF, https://osf.io/ge6vh/). The protocol was amended before data were unblinded (for details see Box A.1 in the Supplementary Materials).

### Participants recruitment

Participants were enrolled between 18th February 2015 and 16th March 2016 through open and remote online recruitment,^14^ with no researcher involvement and minimal participant burden. The study was advertised through paid advertisements on Twitter and Facebook, supplemented by emails and posters within Bupa and the University. Participants were invited to a study conducted in collaboration between the University team and Bupa comparing different features within the BupaQuit app. The differences between conditions were not disclosed. The app could also be found through online searches and on UK app stores. Interested participants were directed to the project website (Figure A.1), with a study information sheet that was also available upon app download, and then to download the app for free.

### Participant eligibility

Participants were eligible if they were (a) UK-based, (b) 18 years or older, (c) smoked daily, (d) wanted to make a serious quit attempt, (e) completed registration, (f) were willing to set a quit date within 2 weeks of registration, (g) agreed to follow-up, (h) agreed to, if invited, confirm abstinent with a personal CO monitor posted to them for free, (i) consented and agreed to Bupa's End User License Agreement (EULA). Criteria (a)–(e) were assessed through a baseline questionnaire. Criteria (f)–(i) were part of consent and app onboarding. Eligibility screening was automated but later supplemented by manual checks based on the unique device ID, name and contact details (24/32, or 75% of duplicate accounts were identified manually).

### Sample size

Given limited information, the effect size estimates were based on SF28 results,^11^ assuming that the control app would be slightly less effective and the intervention app slightly more effective, with predicted success rates of 17% and 25%, respectively (OR = 1.6). This expected difference would be clinically meaningful.^25^ A sample size of 812 would be required to detect this effect in two-tailed analysis with alpha = 0.05 and 80% power. Due to slower recruitment than anticipated, and thus under-recruitment within the time and resources available, the final study sample was 425 participants, which had 51% power to detect the predicted effect. We addressed this limitation by calculating Bayes factors for the abstinence outcomes (see Data Analysis below).^26^

### Bupaquit platform

BupaQuit was developed for iOS and Android by Bupa (www.bupa.com), with the process overseen primarily by the first and sixth author (for details see Box A.2). This involved adapting the original SF28 content,^11^ creating new content and designs to reflect Bupa branding, adding Bupa and University logos and developing a bespoke database. The control version of BupaQuit was developed simultaneously to act as a minimum credible intervention, proving basic functionality that users could expect from a cessation app. The quit plan, and look and feel of the control and intervention versions were identical. The Supplemental Materials provide BupaQuit screenshots (Figure A.2), comparison of SF28, BupaQuit intervention and control on functionality and BCTs^27^ (Table A.1), participant journey through the trial (Figure A.3) and app (Figure A.4). BupaQuit was accessible offline, except for changing the quit date and completing follow-up questionnaires to enable data synchronisation. Participants were free to use the app *ad libitum*, but the app encouraged regular (daily) use through push notifications. Due to study protocol and policy changes in iTunes store on data collection within the apps, no changes or bug fixes to BupaQuit could be made during the trial, except for increasing the size of the control app to match that of the intervention app to minimise differences on download (implemented after 196 users were enrolled).

#### Bupaquit control app

The control app required setting a quit date within 2 weeks of app download, encouraged use of cessation medications, offered minimal support for up to 6 weeks (14 days before the quit date: *pre-quit*, and up to 28 days after the quit date: *post-quit*), including advice on pharmacotherapy, lifestyle changes, daily push-notifications that could be disabled, brief feedback on smoking status, sections ‘about the study’, ‘about the app’, a timeline with progress and tracking of money saved, a meter for momentary cravings (a scale from 0–4^28^), and an option to share the progress on social media.

#### Bupaquit intervention app

In addition to the functionality in the control app, the intervention app included CMTs that were suggested to users reporting ≥1 on the craving meter during *post-quit* app. The CMTs included components from SF28, and were informed by research or theory that suggested potential usefulness at managing cravings: a game promoting distraction,^16,29^ *4Weeks2Freedom* videos presenting self-recorded accounts of smokers trying to quit, which was designed to boost motivation and self-efficacy,^30^ music, audio recordings of guided relaxation routines (e.g. ‘body scan’),^31,32^ descriptions of exercises and activities (e.g. fist clenching, brisk walking^18,33,34^), and motivation boosting tips (e.g. strengthening ex-smoker identity).^20^ The app also offered gamification features (e.g. unlocking of craving aids when engaging with the app), a new piece of brief advice on lifestyle changes that unlock in weeks 2–4, and a longer feedback on smoking status. Some intervention content (e.g. videos or music) was available for free upon additional download.

### Trial procedures

**Figure 1.**
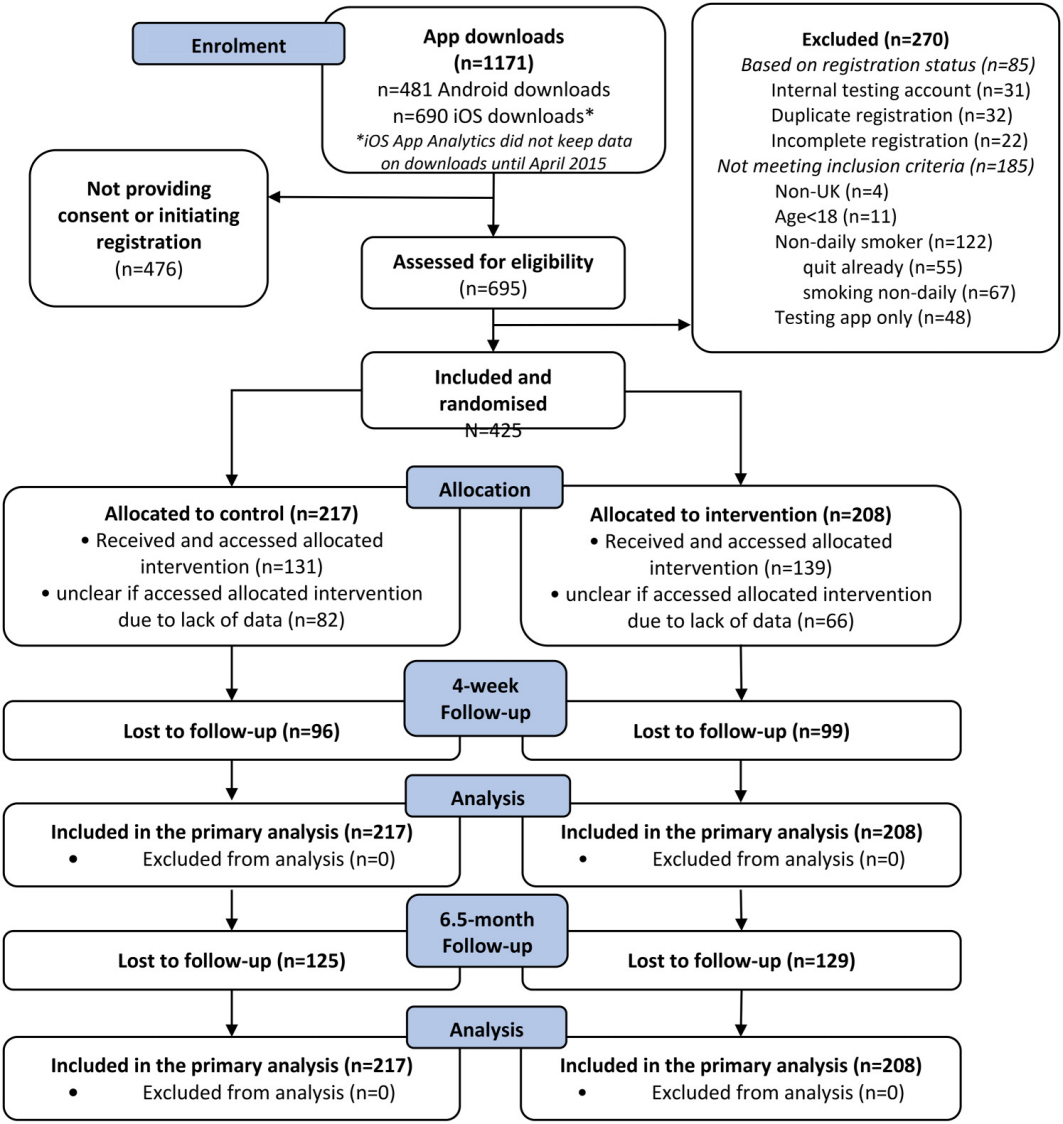
Flowchart of participants in the BupaQuit trial.

 Figure 1 presents the flowchart of participants. After download, participants provided consent and accepted EULA (via tick box), set a quit date, registered and provided contact details, completed baseline and received access to the allocated app version. Participants meeting eligibility criteria were followed-up at 4 weeks and 6.5 months after the final quit date set during their first quit attempt (to account for 2-week grace period following the quit date^35^). Participants were not reimbursed for their participation in the study nor for providing self-reported outcome data, which is in contrast to some previous studies.^8,10^ Reimbursement or incentives were not used in the present study in order to increase external validity and generalisability of the findings to real world settings, where such reimbursement would not be offered. Moreover, as the study was conducted entirely remotely and involved behaviours that participants could have routinely engaged in outside of the study, it was judged, in agreement with the ethics committee reviewing this study, that participant burden and any levels of discomfort from participation were low and not necessitating additional incentives.

Early in the trial we found out that for some participants (*app*-*data-missing*) usage-related data were missing (due to database architecture, these included data on the quit date, operating system, cigarettes smoked per day, and weekly spent). The possible causes were: (i) a failure of synchronisation due to offline use (for seven users the data synchronised with a delay), (ii) interrupted installation, or (iii) not accessing the app after registration. Consequently, in the absence of quit date information, these participants were followed-up at 5 weeks and 7 months since registration. These participants were excluded in sensitivity analyses.

Tables A.2 and A.3a-b outline the schedule of procedures and questionnaires. All assessors were blind to condition allocation. At 4-weeks, the follow-up was via the app (up to three push notifications), e-mail (two emails) and phone (up to four calls). At 6.5 months, the follow-up was via email and phone. The phone follow up only asked about the smoking status. We trialled follow-up through SMS texting, but it was not successful and was discontinued. We also attempted remote biochemical verification of abstinence using personal carbon monoxide monitors developed by Bedfont® Scientific Ltd (COmpact Smokerlyzer®), but this proved to be an infeasible method, with only 15% of CO readings returned from participants reporting abstinence (the majority confirming abstinence) (for details see^36^). Possible reasons could include insufficient contact with participants and lack of reimbursement.^8,12^

### Measures

#### Baseline measures

The baseline survey was mandatory, and collected data on socio-demographic characteristics; smoking, quitting, restriction on phone use and recruitment channel (Table A.2). We recorded operating system (iOS, Android, or Unkown for participants with app-missing-data), and the quit date.

#### Primary and secondary outcomes

The primary outcome was self-reported abstinence in the past 14 days at 4-weeks.^37,38^ Analysis was as per intention to treat, and participants lost to follow up were presumed to have resumed smoking.

Secondary outcomes were (1) 6-month point prevalence (not smoking in the past 7-days) and continuous 6-month abstinence (allowing for smoking of ≤5 cigarettes, and not smoking in the past 7 days);^35^ (2) follow-up channel; (3) app usage (logins, time spent, time/login, proportion of users accessing pre- and post-quit app, accessing craving aids); and (4) satisfaction (only 31 participants provided this data via app or email; the findings are reported in Table A.5).

### Data analysis

Data analyses were conducted by the first author, with no Bupa involvement. Information on group assignment was kept separate from the primary outcome data until an analysis plan was registered on OSF (https://osf.io/vau42/). The primary outcome data were analysed by Fisher's exact test using intention-to-treat (ITT). Relative risk (RR) and 95% confidence intervals were calculated. We also assessed abstinence at short and long term using log-binomial regressions with and without adjustment for baseline characteristics. Analyses of secondary outcomes were conducted using *t*-test and Mann–Whitney *U*-test, and chi-square. All tests were two-sided with alpha initially set to 0.05. Sensitivity analyses were conducted (* denotes pre-registered analyses) that were limited to (a) complete cases*, (b) Users Sample*, (c) *Post-Quit app Users* (as in SF28 analysis^11^). Sidak correction was used for multiple comparisons. Sidak was preferred over Bonferroni correction as the latter has been shown to overcorrect, unnecessarily raising the Type II error rate, while the latter is less conservative while maintaining an acceptable Type I error rate.^39^ We also calculated Bayes factors using an online calculator (https://medstats.github.io/bayesfactor.html). This analysis can distinguish between the likelihood of both the null and alternative hypotheses, and assess whether the data are insensitive.^26,30,40^ We used a uniform distribution with an expected effect size of OR of 1 to 3 versus 1*. We also used a conservative approach with half-normal distribution, with the mode at 0 (no intervention effect), and the standard deviation equal to the expected effect size of OR = 1.6, and other plausible effects of OR = 1.2 and OR = 2.5.

## Results

### Participants

Out of 695 complete registrations, 425 participants met the inclusion criteria (217 were randomised to the control and 208 to the intervention; Figure 1). Participants were 33 years old on average, 45.5% were female, the majority had post-16 education and had previously used cessation support, and 49.3% had a manual occupation (Table 1). Among the trial sample, 34% classified as app-data-missing participants (i.e. they either did not access the app after registration, used it offline, or the data failed to synchronise with the server for other reasons). Except for the Users Sample being slightly older (31 vs 34 years, *p* = 0.01), there was no statistically significant differences on baseline characteristics between participants with and without the app data (Table A.4).

**Table 1.** Baseline characteristics of BupaQuit trial participants.

	Total (*n* = 425)	Intervention (*n* = 208)	Control (*n* = 217)
Age (years); Mean (SD)	32.9 (11.19)	33.05 (10.10)	32.76 (11.40)
Cigarettes smoked per day; Mean (SD)	15.32 (7.17)	15.08 (7.32)	15.58 (7.03)
Weekly spent on cigarettes (GBP)^a^; Mean (SD)	39.92 (50.11)	39.85 (66.08)	40.01 (24.02)
Smokes within 5 min of waking up; % (*N*)	21.4 (91)	19.7 (41)	23.0 (50)
Confidence to stop (1–7); Mean (SD)	4.88 (1.36)	4.98 (1.35)	4.78 (1.37)
Female % (*N*)	45.5 (193)	44.7 (93)	46.1 (100)
Occupation % (*N*)			
Manual	49.2 (209)	51.0 (106)	47.5 (103)
Non-manual	26.4 (112)	25.0 (50)	28.6 (62)
Other (incl. retired, unemployed, student)	24.5 (104)	25.0 (52)	24.0 (52)
Has post-16 yrs qualification; % (*N*)	68.7 (29)	70.2 (146)	67.3 (146)
Time with urges (0–5); Mean (SD)	3.71 (.99)	2.7 (.95)	2.7 (1.05)
Strength of urges (0–5); Mean (SD)	2.81 (.86)	2.75 (.86)	2.8 (.94)
Made an attempt to quit last year; % (*N*)	63.1 (268)	63.9 (133)	62.2 (135)
Stopped smoking for more than 1 week; % (*N*)	76.0 (323)	77.9 (162)	74.2 (161)
Recruitment channel			
Advertisement on Twitter/Facebook	33.9 (144)	33.7 (70)	34.1 (74)
App store searches	36.5 (155)	39.4 (82)	33.6 (73)
Other (email, word of mouth, poster)	29.6 (126)	26.9 (56)	32.3 (70)
Restricted phone access during the day; % (*N*)	23.3 (99)	22.1 (46)	24.4 (53)
Used any cessation aids in the past^b^; % (*N*)			
No aids	19.1 (81)	18.8 (39)	19.4 (42)
Stop smoking services	31.1 (132)	31.2 (65)	30.9 (67)
Medications	52.7 (224)	47.6 (99)	57.6 (125)
E-cigarettes	50.1 (213)	51.4 (107)	48.8 (106)
Apps	20.2 (86)	21.2 (44)	19.4 (42)
Other incl. websites and quitline	16.2 (69)	17.3 (36)	15.2 (33)
Current use of cessation aids ^a,b^; % (*N*)			
No aids	54.2 (150)	59.2 (84)	48.9 (66)
Stop smoking services	5.1. (14)	4.9 (7)	5.2 (7)
Medications	19.9 (55)	14.8 (21)	25.2 (34)
E-cigarettes	26.0 (72)	24.6 (35)	27.4 (37)
Other (incl. apps, websites, quitlines)	6.1 (17)	7.0 (10)	5.2 (7)
Operating system^a^; % (*N*)			
iOS	36.2 (154)	36.1 (75)	36.4 (79)
Android	28.9 (123)	32.2 (67)	25.8 (56)
Unknown	34.7 (148)	31.7 (66)	37.8 (82)
Set Quit Date to Today^a^	68.9 (190)	69.7 (99)	67.4 (91)

^a^ Data available for 277 participants (135 from control and 142 from intervention). The data missing from the remaining participants could be due to failed synchronisation, use of app offline only, or not opening the app after registration.

^b^ Participant could select ‘no aids used’ or select one or more aids.

### Cessation outcomes

The overall abstinence rate was similar between the groups on the primary (13.5% vs 15.7%, *p* = 0.58) and secondary cessation outcomes (sustained 6-month abstinence: 11.1% vs 13.4%; and 6-month point prevalence: 14.4% vs 17.1%, see Table 2). The findings did not change after adjustment for baseline characteristics and in sensitivity analyses. On the primary outcome, the Bayes factor calculated using a uniform distribution supported the null hypothesis (Bu[0, 0, 1.0986] = 0.201). The Bayes factors using the half-normal distribution suggested that the data were insensitive for low effect sizes, but that for OR = 2.5 the data supported the null hypothesis. Conclusions from Bayes factors analyses were similar for the secondary cessation outcomes (see Table 2).

**Table 2.** Abstinence rates at 4 weeks and 6.5 months in BupaQuit trial.

	Intervention	Control			Bayes Factor^a^distribution	
Outcome (all self-reported)	% (*n*/*N*)		*p* ^1^	RR (95% CI) (unadjusted)^2^	Uniform	Half-normal
Continuous abstinence at 4-weeks						
14-day ^FS,^ ^ITT^	13.5 (28/208)	15.7 (34/217)	0.58	0.86 (0.54 to 1.36)	**0.20**	0.64^b^,0.34^c^,0.**19**^d^
14-day ^FS, CC^	25.7 (28/109)	28.1 (34/121)	0.77	0.91 (0.60 to 1.40)	**0.25**	0.73^b^,0.41^c^,0.**23**^d^
14-day ^US, ITT^	14.1 (20/142)	16.3 (22/135)	0.62	0.86 (0.50 to 1.51)	**0.15**	0.47^b^,0.73^c^,0.**24**^d^
14-day ^US, CC^	26.3 (20/76)	28.2 (22/78)	0.86	0.93 (0.56 to 1.56)	0.34	0.82^b^,0.52^c^,0.**30**^d^
14-day ^PQU ITT^	16.5 (18/109)	19.5 (22/113)	0.60	0.85 (0.48 to 1.49)	**0.27**	0.73^b^,0.43^c^,0.**24**^d^
14-day ^PQU CC^	30.0 (18/60)	32.8 (22/67)	0.85	0.91 (0.54 to 1.53)	0.34	0.81^b^,0.52^c^,0.**30**^d^
Abstinence at 6.5-month						
Sustained ^†,^^FS, ITT^	11.1 (23/208)	13.4 (29/217)	0.55	0.83 (0.50 to 1.38)	**0.21**	0.65^b^,0**.**35^c^**,0.19**^d^
Sustained ^FS, CC^	29.1 (23/79)	30.4 (29/92)	0.74	0.92 (0.59 to 1.46)	**0.29**	0.77^b^,0**.**47^c^**,0.27**^d^
Sustained ^US, ITT^	10.6 (15/142)	14.1 (19/135)	0.46	0.75 (0.40 to 1.42)	**0.23**	0.67^b^,0**.**38^c^**,0.21**^d^
Sustained ^US, CC^	26.8 (15/56)	32.8 (19/58)	0.54	0.82 (0.46 o 1.44)	**0.29**	0.74^b^,0**.**45^c^**,0.26**^d^
Sustained ^PQU ITT^	12.8 (14/109)	15.9 (18/113)	0.57	0.81 (0.42 to 1.54)	**0.30**	0.73^b^,0.44^c^,0.25^d^
Sustained ^PQU CC^	31.1 (14/45)	35.3 (18/51)	0.83	0.88 (0.50 to 1.56)	0.36	0.81^b^,0.54^c^,0.**32**^d^
7-day PP ^FS, ITT^	14.4 (30/208)	17.1 (37/217)	0.51	0.85 (0.54 to 1.32)	**0.18**	0.61^b^,0**.32**^c^**,0.17**^d^
7-day PP ^FS, CC^	38.0 (30/79)	40.2 (37/92)	0.88	0.94 (0.65 to 1.38)	**0.29**	0.77^b^,0**.**46^c^**,0.26**^d^
7-day PP ^US, ITT^	14.1 (20/142)	18.5 (25/135)	0.33	0.76 (0.44 to 1.30)	**0.19**	0.62^b^,0**.33**^c^**,0.18**^d^
7-day PP ^US, CC^	35.7 (20/56)	43.1 (25/58)	0.45	0.83 (0.52 to 1.31)	**0.26**	0.70^b^,0**.**41^c^**,0.23**^d^
7-day PP ^PQU ITT^	15.6 (17/109)	21.2 (24/113)	0.30	0.73 (0.42 to 1.29)	**0.20**	0.62^b^,0.**33**^c^,0.**18**^d^
7-day PP ^PQU CC^	37.8 (17/45)	47.1 (24/51)	0.41	0.80 (0.50 to 1.29)	**0.26**	0.70^b^,0.41^c^,0.42^d^

^FS^Full sample eligible at baseline; ^ITT^ Intention-to-treat analysis; ^CC^ Complete case analysis (excluding participants who were not reached at follow-up); ^US^ Users sample (excluding participants with app-data-missing). ^PQ^ Post-quit users (limited to participants who used the app after the quit date, when more features were available, including craving meter and craving aids).

† Sustained abstinence: smoking five cigarettes or less in the past 6 months and not smoking in the past 7 days; PP: point prevalence.

^1^ *p*-value from Fisher's exact test.

^2^ We conducted adjusted analyses of short- and long-term abstinence among the full study sample, which did not affect the results.

a For Bayes factor calculation using uniform distribution (pre-registered), we set the expected effect to be between odds ratio of 1 and 3, versus 1. For Bayes factors calculation using the half-normal distribution (exploratory), the effect sizes used to *specify the* standard deviation of the theory (normal logarithm of ORs) for the half-normal distributions representing the alternative hypotheses were as follows: ^b^OR = 1.2; ^c^OR = 1.6, ^d^OR = 2.5 (Brown et al. 2016; Naughton et al. 2017). The Bayes factors presented in bold mean that the findings supported the null hypothesis, and the rest suggested the data to be insensitive.

### Follow-up rates

At 4-week and 6.5-month, 54.1% and 40.2% participants were contacted, respectively, primarily via the phone (Table 3). There were no statistically significant differences in follow-up rates between the study arms, participants with or without the app data, or across baseline characteristics, except for men being more likely to be contacted at 6 months (*p* = 0.004) (data not reported).

**Table 3.** Follow-up rate, follow-up channels and app usage in BupaQuit trial.

	Intervention	Control	*p*
Follow-up rate at 4 weeks; % (*n*/*N*)	52.4 (109/208)	55.8 (121/217)	0.49
Follow-up channel for primary outcome at 4 weeks; % (*n*/*N*)			
App	7.3 (8)	12.4 (15)	0.58
Email	9.2 (10)	9.9 (12)	
Phone	80.7 (88)	76.0 (92)	
SMS	2.8 (3)	1.7 (2)	
Follow-up rate at 6.5 months; % (*n*/*N*)	38.0 (79/208)	42.4 (92/217)	0.35
Follow-up via phone at 6.5 months; % (*n*/*N*)	77.2 (61/79)	84.8 (78/92)	0.21
Usage data available (during trial only); % (*n*/*N*)	68.3 (142/208)	62.2 (135/217)	0.19
Total logins; Median (IQR) Mean (SD)^a^	4.0 (8.0)	5.0 (9.0)	0.45
	9.6 (14.7)	10.5 (18.0)	0.63
Total time (s)^b^; Median(IQR) Mean (SD)^a^	202.0 (423.3)	209.0 (342.0)	0.54
	401.8 (551.8)	325.8 (418.3)	0.20
Time per login (s)^b^; Median(IQR) Mean (SD)^a^	44.6 (59.9)	32.9 (37.9)	0.01
	64.0 (70.6)	43.5 (40.6)	0.00
App usage classification^c^			
Accessed only pre-quit app	23.2 (33)	16.3 (22)	0.20
Accessed only post-quit app	25.4 (36)	33.3 (45)	
Accessed both pre- and post-quit app	51.4 (73)	50.4 (68)	

^a^ We provide Means to enable comparison with other studies. However, the usage data were skewed and hence we conducted and report results from non-parametric tests comparing usage between the two study arms.

^b^ Total time, excluding registration, from the first use until follow-up. The time spent is an underestimate: (a) data from offline app use save locally on user's device but would not synchronise if users had not accessed the app while being online on a future occasion; and (b) the interaction between an app page and the server occurs when a page is loaded. No further communication with the server occurs until another page is loaded. Hence, it is not possible to identify the exact duration of the last interaction when it ends with exiting the app.

^c^ Only assessed among the sample with usage data available. Pre-quit app use only means that participants set the quit date in the future and accessed only pre-quit content; only the post-quit intervention app offered craving aids.

### App usage

Usage data between intervention and control participants were similar in terms of the login times (median = 4 vs 5, *p* = 0.45; mean = 9.55 vs 10.5, *p* = 0.63), total time spent using the app (median = 202 s vs 209 s, *p* = 0.54; mean = 401.8 vs 325.8, *p* = 0.20), or the proportion of sample accessing only pre-quit content of the app (23.2% and 16.3%) (Table 3). Intervention users tended to spend more time on app per login (median = 44.6 s vs 32.9 s, *p* = 0.01; mean = 64.0 s vs 43.5, *p* = 0.003). Only 48 (23.1% of all intervention participants, 44% among those using BupaQuit post-quit where craving aids were available), accessed any craving aids (median = 3 aids accessed, range: 1–34).

## Discussion

Craving management tools offered within the BupaQuit app had no detectable effect on quit rates. The self-reported quit rates were within the ranges reported in other studies^8,12,14^ and were comparable to those in SF28 study when the analysis was restricted to a similar sample of participants (users who used the app post-quit date).^11^ The engagement levels were comparable to SF28 app,^11^ but were nevertheless relatively low, including with the craving aids, which is a possible explanation for low effectiveness, which echoes findings from similar studies.^41^ The lack of contact with researchers at enrolment might have contributed to suboptimal engagement.^42^ It is also plausible that even with greater engagement, any impact of craving management tools would be too small to be detected, especially over and above the impact of other active components and evidence-based advice offered within the control app version, including setting up the quit date, monitoring of progress and using pharmacotherapy.^43,44^ Further factors that may have contributed to the ineffectiveness of the intervention and low engagement include a lack of in-depth co-design during the development stage, limited adaptively tailored content and exclusion of hybrid components, such as human support.

The methodological challenges encountered during the conduct of this trial point to important feasibility issues facing remote recruitment and retention in smartphone-based smoking cessation interventions, particularly when cessation apps were an emerging technology. The publication of null findings from trials of such apps is important to counter the existing in the literature biases against the null findings (i.e. the file-drawer problem) that can further hamper scientific progress.^45^

### Methodological observations

We conducted open and automated recruitment into an RCT embedded within a cessation app that was available to anyone on UK app stores, which involved the collection of contact details. This enrolment process differed to those used in other studies that included intermediary steps of contacting the researchers or completing additional screening procedures.^8,9,14^ Most users who initiated registration completed baseline with all fields mandatory, and provided plausible contact details. However, policies of app stores may limit what identifiable data could be requested from users, thus affecting study procedures. Furthermore, reliance on the automated screening of app registrations emerged as insufficient. Additionally, most participants were recruited during paid advertisement campaigns. These observations suggest that dedicated budgets and human resources may be required for recruitment, enrolment, data management and follow-up in smartphone-based studies.

Nevertheless, a major challenge for conducting an evaluation of apps through randomised control design, and for recruitment into apps that are live on app stores, is that any informational and promotional materials (e.g. leaflets, but also information on app stores) must conceal the differences between the conditions. This prevents promoting many of the core features offered only within the intervention app version and may result in a recruitment campaign that is less appealing and thus likely to be less effective than one that would actively highlight the intervention features. To improve the reach of recruitment campaigns, future studies could explore diversifying promotional strategies and partnering with national or local organisations to support app promotion within their networks.

Furthermore, the telephone follow-up was the most successful, but it rarely allowed for a longer discussion with participants, while the re-contact via app, email or texting yielded very poor results. This limits the volume of secondary outcome data that can be feasibly collected in such studies. Finally, sensitivity analysis suggests that participants accessing BupaQuit app post-quit, which offered additional features (monitoring and feedback on smoking in both app versions, and craving aids in the intervention), might had higher quit rates than those who set the quit date in the future but never access the app post-quit. Future research should explore and account for the impact that different pre- and post-quit features in stop smoking apps might have on user behaviour and cessation.

### Limitations

First, the study was underpowered to detect the original effect expected, but the Bayes factors suggest it is unlikely that a greater sample would bring support for the alternative hypothesis. Second, only a minority of participants responded to the app or e-mail follow-up, thus providing data on satisfaction. Third, despite the relatively intensive follow-up outside of the app,^37,38,46^ the follow-up rates were falling within the lower end of the spectrum for re-contact rates in other studies.^8,37,47^ The lack of incentives for participation has increased ecological validity of the findings, but it has likely negatively affected both recruitment and follow-up.^8^ Fourth, self-reported abstinence rates tend to overestimate the actual quit rates, although the bias may be lower in remote interventions, and should not differ across study arms.^48,49^ Fifth, we were missing app usage data from a third of participants who met trial eligibility criteria and could not account for this data missingness. Importantly, except for being younger, these participants did not differ from those with complete data, and excluding them from the analyses has not affected conclusions. The burden of joining this study was higher than accessing normal apps on the market but lower than that in previous studies of cessation apps.^8,9,12^ Nevertheless, this is limiting the generalisability of the findings to a wider population of smokers using apps.

### Future directions

Managing cigarette cravings can benefit cessation,^16,18^ and it has been mentioned by smokers as a desired feature of digital interventions.^50,51^ Future research should explore new ways of delivering more engaging and usable CMTs. This could involve utilisation of user-centred approaches^52^ and other research designs, such as Multiphase Optimisation Strategy (MOST;^53,54^), to assess usability and impact of a range of, or a combination of, craving aids, as well as different app architectures and user journeys. It would also need to be ascertained if greater contact with researchers at enrolment could improve engagement and outcomes.

Moreover, smartphone technology and app software have rapidly progressed in the past years, paving ways for new intervention components and tools to support remote smartphone research, including hybrid approaches. These include chatbots powered by artificial intelligence to mirror human–human interaction that might be used to increase engagement with apps^55^ or heart rate monitoring apps that showed early promise for remote verification of abstinence from smoking.^56^ Such developments provide new opportunities for delivering cessation support and should be explored in relation to craving management in the future. Similarly, potential participants now may have greater experience with health-related apps, more digital skills, and different expectations for app functionality and design, which may mean different results in future studies. This is particularly true following the suspension of many face-to-face support services during the COVID-19 pandemic, which has led to service providers pivoting towards digital offers.

## Conclusions

In this pragmatic trial, the addition of craving management tools to the BupaQuit app did not affect cessation, and limited engagement with the app may have contributed to this.

## Supplemental Material

sj-docx-1-dhj-10.1177_20552076211058935 - Supplemental material for Does addition of craving management tools in a stop smoking app improve quit rates among adult smokers? Results from BupaQuit pragmatic pilot randomised controlled trial Click here for additional data file. Supplemental material, sj-docx-1-dhj-10.1177_20552076211058935 for Does addition of craving management tools in a stop smoking app improve quit rates among adult smokers? Results from BupaQuit pragmatic pilot randomised controlled trial by Aleksandra Herbec, Lion Shahab, Jamie Brown, Harveen Kaur Ubhi, Emma Beard, Alexandru Matei and Robert West in Digital Health
