# Supplementary material for: Does addition of craving management tools in a stop smoking app improve quit rates among adult smokers? Results from BupaQuit pragmatic pilot randomised controlled trial
Source: Digit Health. 2021 Nov 23;7:20552076211058935. doi: 10.1177/20552076211058935 (PMC8637712; doi:10.1177/20552076211058935)
Supplement: sj-docx-1-dhj-10.1177_20552076211058935 - Supplemental material for Does addition of craving management tools in a stop smoking app improve quit rates among adult smokers? Results from BupaQuit pragmatic pilot randomised controlled trial [file sj-docx-1-dhj-10.1177_20552076211058935.docx]

**BupaQuit Trial – Supplementary Materials**

***Below is supporting information for manuscript entitled:***

**Does addition of craving management tools in a stop smoking app improve quit rates among adult smokers? Results from BupaQuit pragmatic pilot randomised controlled trial.**

**Authors:** Aleksandra Herbec* ^a,b,c^ PhD, Lion Shahab^a,c^, PhD, Jamie Brown^a,c,d^, PhD, Harveen Kaur Ubhi^a,c^, PhD, Emma Beard,^a,c,d^, PhD, Alexandru Matei^e,f^, PhD, Robert West^a,c^, PhD.

*** Corresponding Author:** Aleksandra Herbec, Department of Behavioural Science and Health, Rm 215, University College London, 1-19 Torrington Place, London WC1E 6BT, UK.

E-mail: [a.herbec@ucl.ac.uk](mailto:a.herbec@ucl.ac.uk)

Twitter: @AHerbec

^a^ Department of Behavioural Science and Health, University College London, 1-19 Torrington Place, London, WC1E 6BT, UK,

^b^ UCL Centre for Behaviour Change, Department of Clinical, Educational and Health Psychology, University College London, 1-19 Torrington Place, London, WC1E 6BT, UK

^c^ UCL Tobacco and Alcohol Research Group (UTARG), University College London,

1-19 Torrington Place, London, WC1E 6BT, UK

^d^ Department of Clinical, Educational and Health Psychology, University College London,

1-19 Torrington Place, London, WC1E 6BT, UK

^e^ Bupa Centre Medical, Bupa, 15-19 Bloomsbury Way, London, WC1A 2BA

^f^ Department of Computer Science, University College London, London, WC1E 6BT, UK

**Trial registration:** ***ISRCTN10548241***. Registered prospectively on 17^th^ February 2015.

**OSF**: <https://osf.io/ge6vh/> DOI 10.17605/OSF.IO/GE6VH

Contents

[Box A.1: Deviation from the original protocol 2](#_Toc506373247)

[Box A.2: BupaQuit app platform development and data management 3](#_Toc506373248)

[Figure A.1: BupaQuit project website 5](#_Toc506373249)

[Figure A.2: Screenshots of SF28 and BupaQuit app 6](#_Toc506373250)

[Figure A.3: Participant journey through BupaQuit trial and app 7](#_Toc506373251)

[Figure A.4: User Journey through BupaQuit app for returning users 8](#_Toc506373252)

[Table A.1: Comparison of BupaQuit Intervention (Interv), Control (Contr) and SF28 apps on functionality and Behaviour Change Techniques (BCTs) 9](#_Toc506373253)

[Table A.2: Schedule of procedures and measurements in BupaQuit trial. 12](#_Toc506373254)

[Table A.3a-b: Baseline and follow-up questionnaires in BupaQuit Trial. 13](#_Toc506373255)

[Table A.4: Baseline characteristics of trial participants with and without missing app data 15](#_Toc506373256)

[Table A.5: Satisfaction ratings of BupaQuit app. 16](#_Toc506373257)

# Box A.1: Deviation from the original protocol

| **Deviation from the original protocol and reasons** |
| --- |
| Since the trial registration, few changes were made to the original protocol, which were implemented before the data was unblinded. The changes were explained on the updated ISRCNT registration (link removed for blinding), with details on [Ope](https://osf.io/fqaz2/)n Science Framework (link removed for blinding)   - The most important was the change to the original primary outcome, as due to low rate of returning of CO readings, the primary outcome was set to self-reported abstinence. - The other changes included changes to:  1. The inclusion criteria (due to missing data on cigarettes smoked per day among a sample of participants the requirement of smoking min 5 cigarettes per day was removed, and replaced with daily smoking). 2. The secondary outcome measures (removal of biochemical verification of abstinence at 6-month follow-up, and reporting self-reported data only). |

# Box A.2: BupaQuit app platform development and data management

| **Further information about BupaQuit platform development and data management** |
| --- |
| *Platform development*  The development of BupaQuit app was iterative and was overseen by the authors and SF28 developers. First, Bupa purchased a non-exclusive, perpetual licence to the Smokefree 28 (SF28) source code and content (more information about SF28 app, its content and theoretical underpinnings can be found here: [www.smokefree28.com](http://www.smokefree28.com) and [[8](#_ENREF_8)]). SF28 is underpinned by the PRIME Theory of motivation [[29](#_ENREF_29)] which draws together diverse aspects of motivation including personal rules, self-control and momentary influences of desires and impulses [[14](#_ENREF_14), [29](#_ENREF_29)]. SF28 supports users to set a clear goal (a quit date), monitor their progress towards abstinence and offers tools supporting quitting, such as advice on medications, a distracting game and inspirational videos from smokers trying to quit [[14](#_ENREF_14)]. Second, the content of SF28 was reviewed by the first author and a new user flow was created to focus the functionality of BupaQuit Intervention app on craving monitoring and management. Any new content required was drafted by the first author and by Bupa members. Third, the app design was adapted to reflect Bupa branding. An externally contracted company (Jam, www.jam.co.uk) provided expertise in design and user experience, and created the final app designs and user journeys based on the specifications provided and trial requirements. Finally, internal user testing was conducted regularly during app development to assess functionality, usability, data collection, and reminder settings. A usability testing session was organized at the University among novice users to identify any usability issues and bugs, and final adjustments were made (e.g. adding app tutorial, larger fonts on some instructions, clearer instructions).  The code repository used for source code was Github. App Hosting was in Microsoft Azure (for testing), and Rackspace (for production/release and maintenance during the study). |
| *Data management*  Data management and sharing with the researchers at the university was governed by a bespoke Data Licence Agreement, according to which anonymised data from BupaQuit were shared with the study PI and his team for further processing, independent analysis, and dissemination.  **Box A.2 (cont.):** BupaQuit app platform development and data management  *Data management (cont.)*  Upon download and registration via the app, participants’ email address was locked (by assigning to it a unique study ID) to study condition (intervention or control) to minimise contamination. This study ID was used to label any subsequent data collected for that user within and outside of the app.  Additionally, a unique, persistent device ID (UID) was generated upon first registration and used to detect additional registrations from the same device.  Data collected through the app were saved locally on participants’ phones and transferred during online app use to password-protected database servers based in the EU managed by Bupa. Anonymised data were shared with the researchers at the university, and were initially visualised and managed in Tableau® (a data visualisation software) by the first author, and Excel spreadsheets were generated with each participant’s data linked by their study IDs. Primary and secondary outcome data collected outside of the app were entered manually into separate password-protected Excel spreadsheets. As a final step, all relevant and anonymised trial data from baseline, app use, and follow-ups were merged into an Excel file using participants’ study IDs. Data on group assignment was kept separate from the outcome data until an analysis plan and commands to run the analysis had been approved. |

# Figure A.1: BupaQuit project website

BupaQuit project website that included links to the app stores, the complete study information sheet as well as End User Agreement for app use. The website was retired on November 1^st^ 2016.


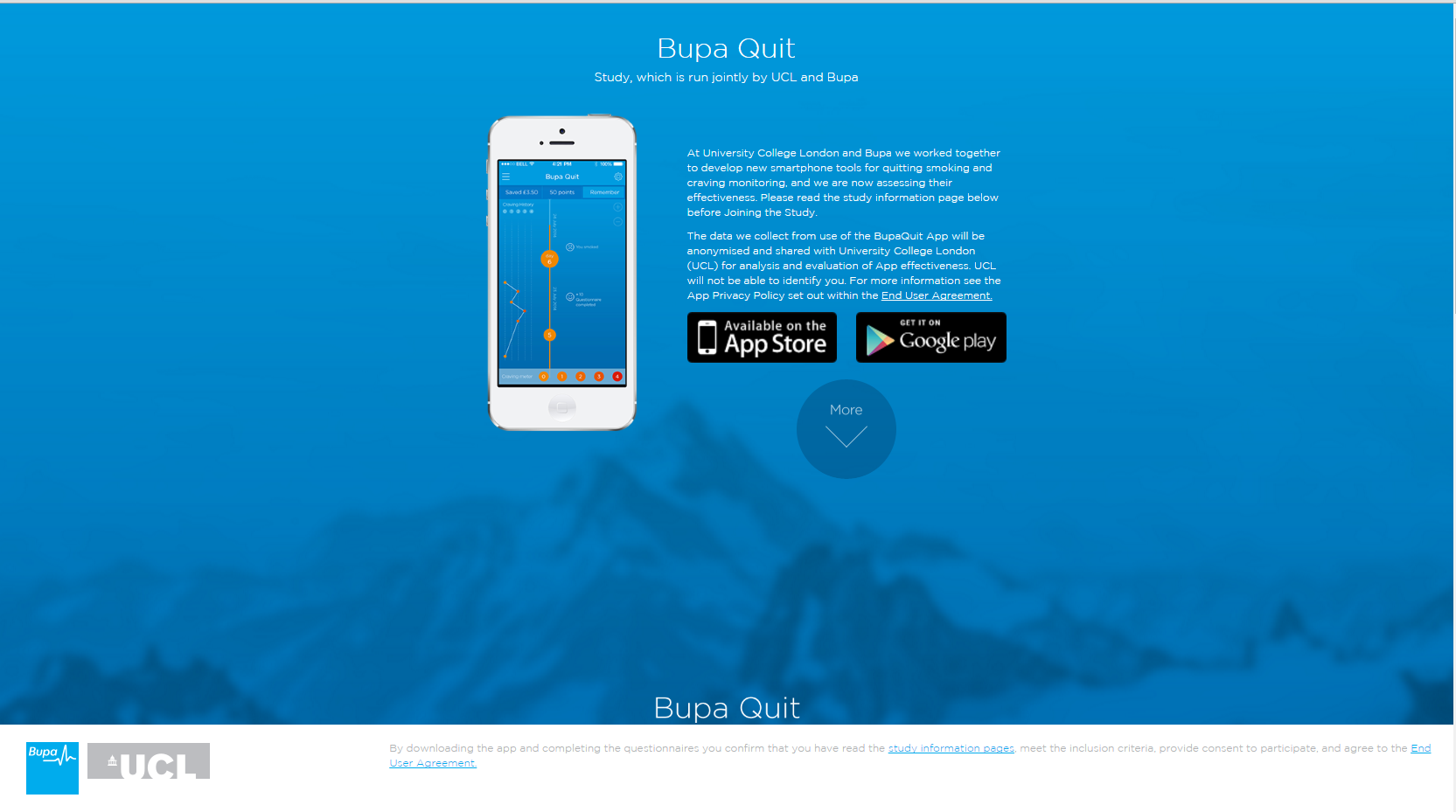


# Figure A.2: *Screenshots of SF28 and BupaQuit app*

Screenshots of SF28 app (dashboard) and of BupaQuit, highlighting the latter’s key functions of BupaQuit app


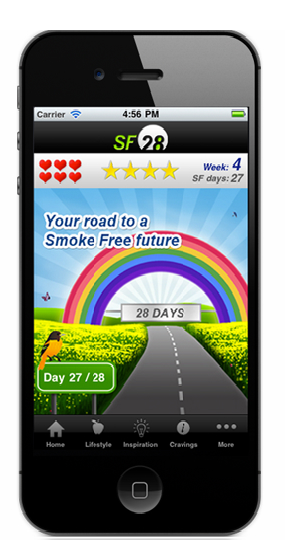

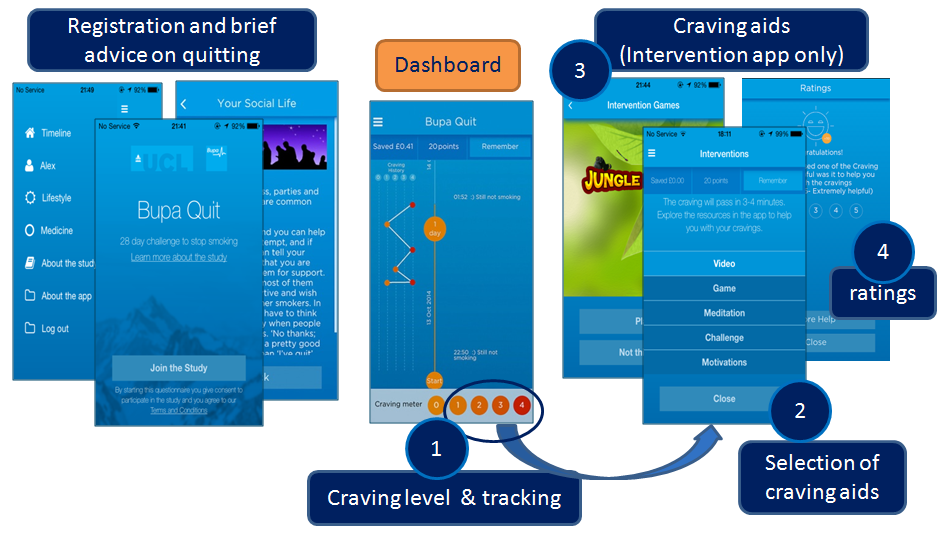


**Adaptation**

# Figure A.3: Participant journey through BupaQuit trial and app


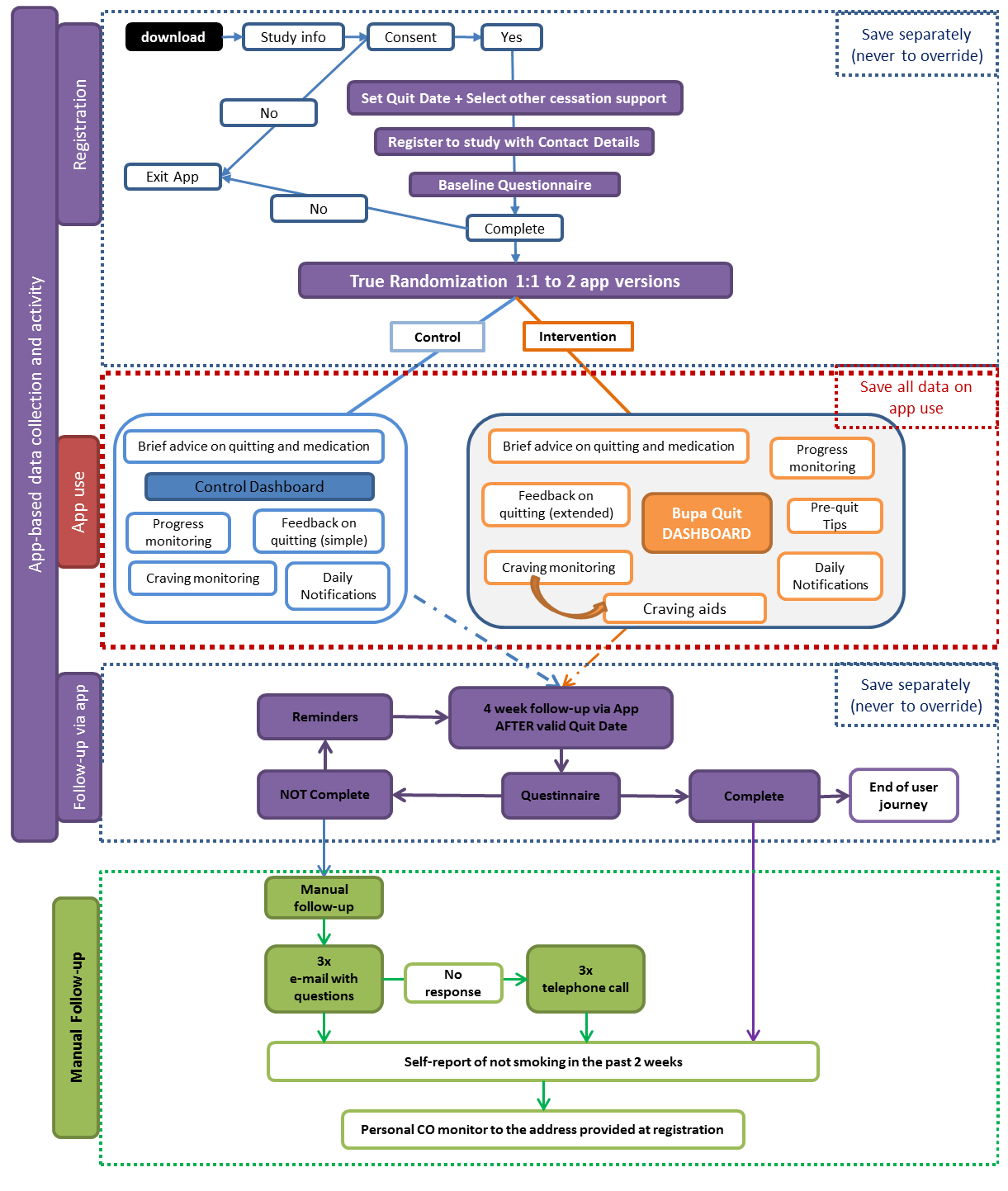


# Figure A.4: User Journey through BupaQuit app for returning users

#
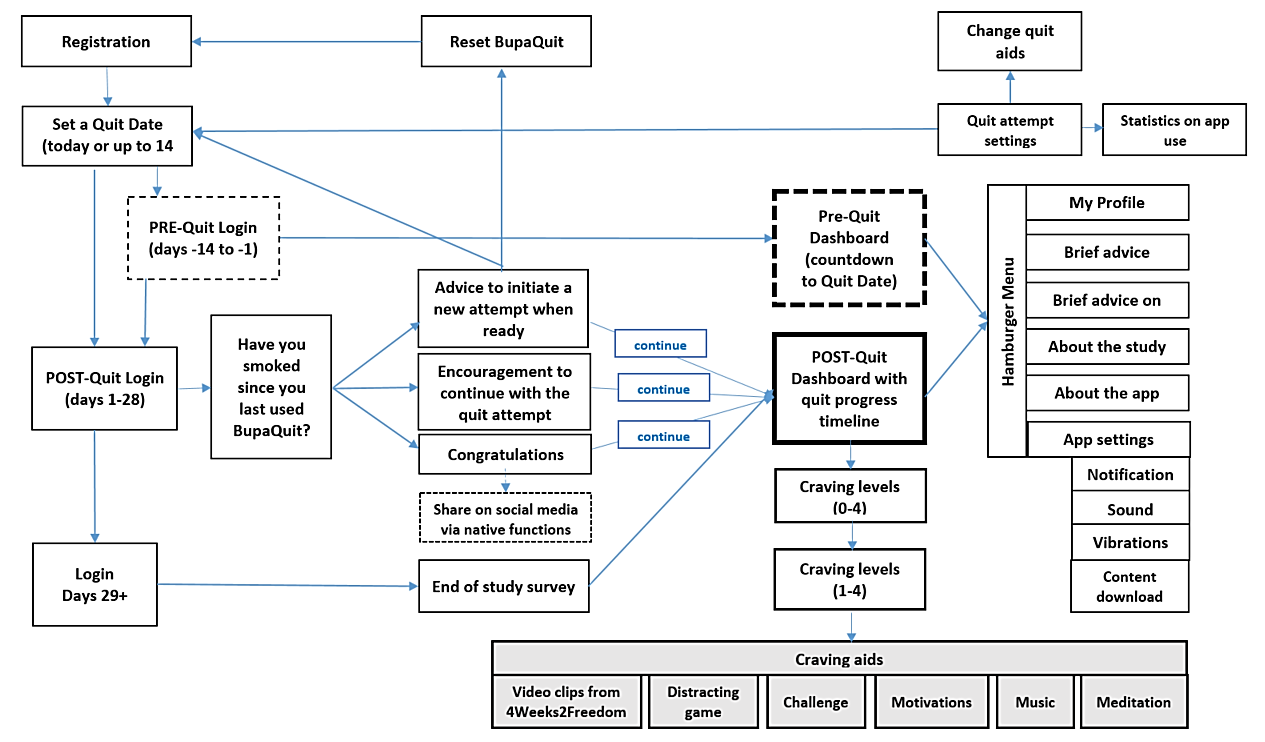


# Table A.1: Comparison of BupaQuit Intervention (Interv), Control (Contr) and SF28 apps on functionality and Behaviour Change Techniques (BCTs)

| **Feature** | **BCTs**  **based on V1 Taxonomy^[[1]](#footnote-1)^** | **BCT**  **from smoking taxonomy^[[2]](#footnote-2)^** | **SF28** | **Bupa Quit** | |  |
| --- | --- | --- | --- | --- | --- | --- |
|  |  |  |  | Interv | Contr |  |
| Registration and Setting of the Quit Date |  |  |  |  |  |  |
| Information about BupaQuit Challenge and app | N/A  Information about natural consequences (5.1-5.3) | RC4 (explain expectations regarding treatment programme) | Yes | Yes | Yes |  |
| Advice on cessation medication | Pharmacological support (11.1) | A1 (advice on stop-smoking medication) | Yes | Yes | Yes |  |
| Identifying / recording the main motivation to quit | Information about social and environmental consequences (5.3) | BM9 (reasons for wanting and not wanting to stop) | - | Yes | Yes |  |
| Setting up of the quit date | Goal setting (behaviour 1.1, outcome 1.3)  Conserving mental resources (11.3)  Commitment (19.1) | BS4 (facilitate goal setting)  BS10 (advise on conserving mental resources)  BM6 (prompt commitment from the client there and then) | Yes | Yes | Yes |  |
| Monitoring of days smoke-free and feedback | Self-monitoring of behaviour (2.2) | BS6 (prompt self-recording) |  |  |  |  |
| Simple positive feedback on not smoking | Feedback on behaviour (2.2) | BM3 (provide feedback on current behaviour)  BM2 (boost motivation and self-efficacy),  BM7 (provide rewards contingent on effort or progress) | Yes | Yes | Yes** |  |
| Daily tips on craving management for non-smokers | Reduce negative emotions (11.2) | BS2, RC6 (provide information on withdrawal symptoms),  RC10 (provide reassurance) | Yes | Yes | - |  |
| Feedback on smoking (incl. advice to reset quit attempt after the 3^rd^ lapse) | Feedback on behaviour (2.2)  The discrepancy between current behaviour and goal (1.6)  Social support (unspecified, 3.1) | BM23 (feedback)  BS1 (Facilitate relapse prevention and coping)  RC10 (provide reassurance) | Yes | Yes | Yes |  |
| Resetting the app after 3^rd^ lapse | N/A | N/A | Yes | - | - |  |
| **Table A.1 (cont)**: Comparison of BupaQuit Intervention (Interv), Control (Contr) and SF28 apps on functionality and Behaviour Change Techniques (BCTs) | | | | | | |
| **Feature** | **V1 BCT Taxonomy ^1^** | **Smoking BCT Taxonomy ^2^** | **SF28** | **Inter** | **Contr** |  |
| Daily reminders to use the app | N/A | N/A | Yes | Yes | Yes |  |
| Tracking of money savings | Feedback on outcomes of behaviour (2.7) | BM3 (provide feedback on current behaviour) | Yes | Yes | Yes |  |
| Generic Advice |  |  |  |  |  |  |
| Pre-quit tips | Information about antecedents (4.2)  Restructuring the physical environment (12.1)  Restructuring the social environment (12.2)  Avoidance/reducing exposure to cues for the behaviour (12.3)  Verbal persuasion about capability (15.3)  Identity associated with changed behaviour (13.5) | A1 (advice on stop-smoking medications),  A2 (advise on/facilitate use of social support), BM1 (provide information on consequences of smoking and smoking cessation),  BM2 (boost motivation and self-efficacy), BM8 (strengthen ex-smoker identity),  BM10 (explain the importance of abrupt cessation) | Yes | Yes | - |  |
| Brief advice on lifestyle changes and social support | Information about antecedents (4.2)  Restructuring the physical environment (12.1)  Restructuring the social environment (12.3)  Avoidance/reducing exposure to cues for the behaviour (12.3)  Verbal persuasion about capability (15.3)  Information about natural consequences (5.1-5.3) | A2 (advise on/facilitate use of social support),, BS7 (advise on changing routines), BS8 (advice on environmental restructuring), BS11 (advise on avoiding social cues for smoking) | Yes* | Yes* | Yes*** |  |
| Advice to think of oneself as a non-smoker | Identity associated with changed behaviour (13.5) | BM8 (strengthen ex-smoker identity) | Yes | Yes | Yes*** |  |
| Information on stop smoking medication | Pharmacological support (11.1) | A1 (advise on stop-smoking medication) | Yes | Yes | Yes |  |
| About the study and the App | Credible source (9.1) | N/A | - | Yes | Yes |  |
| Cravings – monitoring and management |  |  |  |  |  |  |
| Tools assessing momentary cravings | Self-monitoring of outcomes of behaviour (2.4) | BS6 (prompt self-recording)  R14 (assess withdrawal symptoms) | - | Yes | Yes |  |
| Feedback on craving levels | Feedback on outcomes of behaviour (2.7) | R14 (assess withdrawal symptoms)  RC6 (provide information on withdrawal symptoms) | - | Yes | - |  |
| Brief advice on craving management (use NRT) | Pharmacological support (11.1) | A1 (advise on stop-smoking medication) |  |  |  |  |
| Craving management tools | Reduce negative emotions (11.2) |  | Yes | Yes^§^ | - |  |
| Videos diaries of smokers (4Weeks2Freedom) | Reduce negative emotions (11.2)  Information about natural consequences (5.1-5.3)  Social comparison (6.2) | BM2 (boost motivation and self-efficacy),  BM5 (provide normative information about others’ behaviour and experiences) | Yes | Yes | - |  |
| **Table A.1 (cont)**: Comparison of BupaQuit Intervention (Interv), Control (Contr) and SF28 apps on functionality and Behaviour Change Techniques (BCTs) | | | | | | |
| **Feature** | **V1 BCT Taxonomy^1^** | **Smoking BCT Taxonomy ^2^** | SF28 | Inter | Contr |  |
| Relaxation music | Reduce negative emotions (11.2) | BS2 (facilitate relapse prevention and coping) | - | Yes | - |  |
| Meditation | Reduce negative emotions (11.2) | BS2 (facilitate relapse prevention and coping) | - | Yes | - |  |
| Motivation boosters# | Information about natural consequences (5.1-5.3)  anticipated regret (5.5) Information about emotional consequences (5.6)  Self-talk (15.4)  Verbal persuasion about capability (15.1)  Comparative imagining of future outcomes (9.3) | BM1 (provide information on consequences of smoking and smoking cessation)  BM2 (boost motivation and self-efficacy)  BM5 (provide normative information about others’ behaviour and experiences)  BM8 (Strengthen ex-smoker identity),  RC10 (provide reassurance) | Yes | Yes^¥^ | - |  |
| ‘Challenges’## | Distraction (12.4)  Avoidance/reducing exposure to cues for the behaviour | BS1 (facilitate barrier identification and problem-solving)  BS2 (facilitate relapse prevention and coping) | Yes | Yes^¥^ | - |  |
| Game aiding distraction | Distraction (12.4) | BS2 (facilitate relapse prevention and coping) | Yes | Yes | - |  |
| Gamification features | N/A | N/A | - | Yes | Some |  |
| Simple Badges on quit progress | Non-specific rewards (10.3) | BM7 (provide rewards contingent on effort or progress) | - | Yes | - |  |
| Collecting points on App use | N/A | N/A | - | Yes | Yes |  |
| Points unlocking craving management features | N/A | N/A | - | Yes | - |  |
| Statistics on App use with focus on use of craving management features) | N/A | N/A | - | Yes | - |  |
| Information about the team behind BupaQuit | Credible Source (9.1) | RC1 (build general rapport) | - | Yes | Yes |  |

*advice updated weekly

**shorted feedback

***advice provided for the first week was never updated.

# text-based advice

§ content accessible only to those reporting having cravings

## text-based advice and instructions on physical exercises, muscle tensing, relaxation, distraction

¥ based on SF28 content and advice, but with modifications and extension

# Table A.2: Schedule of procedures and measurements in BupaQuit trial.

| **Procedure/assessment** | **Always accessible on the project website** | **S1:**  **Initial App visit** | **S2:**  **4-week post-quit date follow-up** | **S4:**  **6-month post-quit date follow up** |
| --- | --- | --- | --- | --- |
| **Procedures** |  |  |  |  |
| **Show complete Information Sheet** | X | X |  |  |
| **Show End User Licence Agreement** | X | X |  |  |
| **Provide contact details to the research team** | X | X |  |  |
| **Obtain consent** |  | X |  |  |
| **Registration and obtain contact details** |  | X |  |  |
| **Randomisation** |  | X |  |  |
| **Email reminders and follow-up** |  |  | X | X |
| **Telephone follow-up** |  |  | X | X |
| **CO personal monitor postage** |  |  | X |  |
| **Assessment** |  |  |  |  |
| **Demographic information** |  | X |  |  |
| **Smoking history** |  | X |  |  |
| **Dependence levels (HSI)** |  | X |  |  |
| **Smoking abstinence** |  |  | X | X |
| **Urges to smoke** |  | X |  |  |
| **Prior use of cessation medication** |  | X |  |  |
| **Prior use of additional support** |  | X |  |  |
| **Use of cessation aids** |  | X | X |  |
| **CO monitoring test^#^** |  |  | X |  |

###

# Table A.3a-b: Baseline and follow-up questionnaires in BupaQuit Trial.

**Table A.3a: Baseline Questionnaire**

| Q # | **Question** | **Answer options** |
| --- | --- | --- |
|  |  |  |
| 1 | Gender | Man / Woman |
| 2 | Age | Enter free text |
| 3 | Where do you live now? | UK/ Other |
| 4 | Employment status | Manual setting  Non-manual setting  Currently unemployed/retired  Full-time student  Other |
| 5 | Are you free to use your smartphone throughout the day? (e.g. choose No if work regulations limit your access) | Yes / No |
| 6 | Do you have post 16 yrs qualification? (e.g. A-levels, a degree) | Yes / No |
| 7 | Ever used any of these to try to quit smoking in the past? (select all that apply) | Medications (e.g. NRT)  NHS stop smoking service  Quitline / other counselling  Other Apps  Websites  E-cigarettes  Other  None |
| 8 | Made an attempt to quit last year? | Yes / No |
| 9 | Ever stopped smoking for more than a week? | Yes / No |
| 10 | Do you currently smoke? | Yes, daily  Yes, but not daily  No, I already quit |
| 13 | How often did you experience urges to smoke in the past 24 hours? | Not at all  A little of the time  Some of the time  A lot of the time  Almost always  All the time |
| 14 | Only ask if Q13>0.  How strong were those urges to smoke? | (1) ‘slight’  (2) ‘moderate’  (3) ‘strong’  (4) ‘very strong’  (5) ‘extremely strong’ |
| 15 | How soon after you wake up do you smoke your first cigarette? | Within 5 minutes  6-30 minutes  31-60 minutes  More than 60 minutes |
| 16 | Why joined the study | To make a serious quit attempt  I’m just testing the App  Other |
| 17 | Only ask if Q16=Yes  Confidence to quit this time | Scale from 1-7 |
| 18 | How did you learn about this study? | E-mail or poster about the study  Word of mouth (within Bupa)  Word of mouth (outside Bupa)  App store  Other (please specify – free text) |
|  |  |  |
| **Table A.3a (cont.)**: Baseline Questionnaire | | |
| Q # | **Question** | **Answer options** |
| 19 | Current use of other quitting aids (please select all that apply): | Medications (e.g. NRT)  NHS stop smoking service  Quitline / other counselling  Other Apps  Websites  E-cigarettes  Other  None |
| 20 | Current number of cigarettes smoked per day: | Enter |
| 21 | Weekly spend on cigarettes: | Enter |
| 22 | Your main motivation to quit: | (1) To improve my health or fitness  (2) To save money  (3) For my children or loved ones  (4) Not to smell of cigarettes  (5) For healthy looking skin and teeth  (6) To be less stressed or sleep better  (7) Other (free text) |

**Table A.3b:** Follow-up Questionnaires

(*=questions asked via app and email)

| **#** | **Question** | **Answers** |
| --- | --- | --- |
|  | **4-week follow-up (Questions asked via the BupaQuit App and e-mail)** | |
| 1 | Did you smoke at all in the past 2 weeks? | Yes/No |
| 2 | How helpful was the App in helping you to manage cravings? (1-not at all, 5- very helpful)* | (1-not at all, 5- very helpful) |
| 3 | Would you recommend the App to others?* | Yes/No |
| 4 | Would you use the App in the future, if needed?* | Yes/No |
|  | **6-months follow-up** | |
| 5 | Have you smoked at all in the past 6 months (except for the first 2 weeks since the quit date) | 1. No, not even a puff 2. Yes, between 1 and 5 cigarettes 3. Yes, more than 5 cigarettes |
| 6 | Did you smoke any cigarettes in the past 7 days? | Yes/No |

# Table A.4: Baseline characteristics of trial participants with and without missing app data

|  | **Total**  **(n=425)** | **App-Missing Data**  **(n=148)** | **Complete**  **Data**  **(Users Sample)**  **(n=277)** | ***p*** |
| --- | --- | --- | --- | --- |
| **Intervention arm, %(N)** | 48.9 (208) | 44.6 (66) | 51.3 (142) | .19 |
| **Age (years) Mean (SD)** | 32.9 (11.19) | 31.0 (10.3) | 33.9 (11.5) | .01 |
| **Smokes within 5min of waking up % (N)** | 21.4 (91) | 19.6 (29) | 22.4 (62) | .50 |
| **Confidence to stop (1-7) Mean (SD)** | 4.88 (1.36) | 4.8 (1.3) | 4.9 (1.4) | .61 |
| **Female % (N)** | 45.5 (193) | 50.7 (75) | 42.6 (118) | .11 |
| **Occupation % (N)** |  |  |  |  |
| Manual | 49.2 (209) | 51.4 (76) | 48.0 (133) | .72 |
| Non-manual | 26.4 (112) | 26.4 (39) | 26.4 (73) |  |
| Other, incl. retired, unemployed, student | 24.5 (104) | 22.3 (33) | 25.6 (71) |  |
| **Has post-16 yrs qualification % (N)** | 68.7 (292) | 68.9 (102) | 68.6 (190) | .95 |
| **Time with urges (0-5) Mean (SD)** | 2.7 (1.0) | 2.7 (1.0) | 2.7 (1.0) | .51 |
| **Strength of urges (0-5) Mean (SD)** | 2.8 (.9) | 2.8 (.9) | 2.8 (.9) | .86 |
| **Made an attempt to quit last year % (N)** | 63.1 (268) | 60.8 (90) | 64.3 (178) | .48 |
| **Stopped smoking for more than 1 week % (N)** | 76.0 (323) | 75.0 (111) | 76.5 (212) | .72 |
| **Recruitment channel** |  |  |  |  |
| Advertisement on Twitter/Facebook | 33.9 (144) | 27.0 (40) | 37.5 (104) | .09 |
| App store searches | 36.5 (155) | 40.5 (60) | 34.3 (95) |  |
| Other (email, word of mouth, poster) | 29.6 (126) | 32.4 (48) | 28.2 (78) |  |
| **Restricted phone access during the day % (N)** | 23.3 (99) | 21.6 (32) | 24.2 (67) | .55 |
| **Used any cessation aids in the past^#^ % (N)** |  |  |  |  |
| **No aids** | 19.1 (81) | 22.3 (33) | 17.3 (48) | .21 |
| **Stop smoking services** | 31.1 (132) | 28.4 (42) | 32.5 (90) | .38 |
| **Medications** | 52.7 (224) | 50.0 (74) | 54.2 (150) | .41 |
| **E-cigarettes** | 50.1 (213) | 48.6 (72) | 50.9 (141) | .66 |
| **Apps** | 20.2 (86) | 18.9 (28) | 20.9 (58) | .62 |
| **Other incl. websites and quitline** | 16.2 (69) | 12.2 (18) | 18.4 (51) | .10 |

*Note*: The table presents baseline characteristics for intervention and control participants. The exact p-values are presented (results of t-test for continuous variables, and chi-square for categorical). None of the values met threshold for significance after Sidak adjustment for multiple comparisons. # Participants could select one or more answers.

# Table A.5: Satisfaction ratings of BupaQuit app.

|  | | **Intervention** | | **Control** | | ***p*** |
| --- | --- | --- | --- | --- | --- | --- |
| **Satisfaction ratings for BupaQuit, % (n/N)** | |  | |  | |  |
| App being helpful with managing cravings (1-5), Mean (SD)^a^ | 3.3 (1.5) | | 2.8 (1.5) | | .33 | |
| Would use the app in the future, % (n/N)^b^ | 83.3 (10/12) | | 78.9 (15/19) | | .76 | |
| Would recommend the app to a friend, % (n/N) | 83. 3 (10/12) | | 78.9 (15/19) | | .76 | |

*Note*: Satisfaction with app was assessed through: (i) app helpfulness to manage cravings (1-not at all, 5- very helpful), (ii) interest to use in the future (yes/no), (iii) recommending app to others (yes/no) [[45](#_ENREF_45)]. To limit burden, these questions were only asked via app and email. The data were provided by 31 participants in total, during the eligible follow-up period at 4 weeks (26 participants provided the data via the app - three of these participants reported data on primary outcome first via phone or email before completing the follow-up survey via the app; and 5 via email).

1. Michie S, Richardson M, Johnston M, Abraham C, Francis J, Hardeman W, Eccles MP, Cane J, Wood CE. (2013). The Behavior Change Technique Taxonomy (v1) of 93 hierarchically clustered techniques: building an international consensus for the reporting of behavior change interventions, Annals of Behavioral Medicine, 2013;46(1): 81-95. doi: 10.1007/s12160-013-9486-6 [↑](#footnote-ref-1)
2. Michie S, Hyder, N., Walia, A. Wes,t, R. (2011). Development of a taxonomy of behaviour change techniques used in individual behavioural support for smoking cessation. *Addictive Behaviors*, 36(4), 315-319 [↑](#footnote-ref-2)
